# Experimental *Bothrops atrox* Envenomation: Blood Plasma Proteome Effects after Local Tissue Damage and Perspectives on Thromboinflammation

**DOI:** 10.3390/toxins14090613

**Published:** 2022-09-01

**Authors:** Joeliton S. Cavalcante, Ingrid Mayara da Cunha Brito, Laudicéia Alves De Oliveira, Luciana Curtolo De Barros, Cayo Almeida, Bruno Cesar Rossini, Duaran Lopes Sousa, Renata Sousa Alves, Roberta Jeane Bezerra Jorge, Lucilene Delazari dos Santos

**Affiliations:** 1 Graduate Program in Tropical Diseases, Botucatu Medical School (FMB), São Paulo State University (UNESP), Botucatu 18618-687, Brazil; 2 Center for the Study of Venoms and Venomous Animals (CEVAP), São Paulo State University (UNESP), Botucatu 18618-687, Brazil; 3 Center of Mathematics, Computing Sciences and Cognition, Federal University of ABC, São Paulo 09210-580, Brazil; 4 Biotechnology Institute (IBTEC), São Paulo State University (UNESP), Botucatu 18618-687, Brazil; 5 Department of Chemical and Biological Sciences, São Paulo State University (UNESP), Botucatu 18618-687, Brazil; 6 Department of Clinical and Toxicological Analysis, Federal University of Ceará, Fortaleza 60430-160, Brazil; 7 Drug Research and Development Center, Federal University of Ceará (UFC), Fortaleza 60430-275, Brazil; 8 Department of Physiology and Pharmacology, School of Medicine, Federal University of Ceará (UFC), Fortaleza 60430-140, Brazil; 9 Graduate Program in Research and Development (Medical Biotechnology), Botucatu Medical School (FMB), São Paulo State University (UNESP), Botucatu 18618-687, Brazil

**Keywords:** snake venom, *Bothrops atrox*, edema, proteomic analysis, inflammation, thromboinflammation

## Abstract

The clinical manifestations of *Bothrops atrox* envenoming involve local and systemic changes, among which edema requires substantial attention due to its ability to progress to compartmental syndromes and sometimes cause tissue loss and amputations. However, the impact of edema on the poisoned body’s system has not been explored. Thus, the present study aimed to explore the systemic pathological and inflammatory events that are altered by intraplantar injection of *B. atrox* venom in a mouse model through hematologic, lipidic, and shotgun proteomics analysis. Plasma samples collected showed a greater abundance of proteins related to complement, coagulation, lipid system, platelet and neutrophil degranulation, and pathways related to cell death and ischemic tolerance. Interestingly, some proteins, in particular, Prdx2 (peroxiredoxin 2), Hba (hemoglobin subunit alpha), and F9 (Factor IX), increased according to the amount of venom injected. Our findings support that *B. atrox* venom activates multiple blood systems that are involved in thromboinflammation, an observation that may have implications for the pathophysiological progression of envenomations. Furthermore, we report for the first time a potential role of Prdx2, Hba, and F9 as potential markers of the severity of edema/inflammation in mice caused by *B. atrox*.

## 1. Introduction

In the Amazonian region, *Bothrops atrox* is responsible for the highest number of human envenomation and represents the greatest loss due to the severity of the cases and the delay between the accident and the antivenom administration [1,2]. *B. atrox* snake venom has shown high variability; however, snake venom metalloproteinases (SVMPs), snake venom serine proteases (SVSPs), and phospholipases A_2_ are the main toxins of venom, corresponding to most of its composition. L-amino acid oxidase (LAAO), cysteine-rich secretory protein (CRiSP), C-type lectins, and C-type lectin-like (CTL/SNACLEC), disintegrin (DISI) and natriuretic peptides (NP), including vasoactive peptides, bradykinin potentiating and inhibitory peptides also composes this snake venom [3,4]. 

Snake venoms are a complex mixture of proteins, peptides, enzymes, and other components that causes a wide range of manifestations in the bite site, such as pain, edema, hematoma, bleeding, blisters, secondary infection, cellulitis, lymphadenopathy, necrosis, abscess, compartment syndrome, and can also cause systemic manifestations, such as hemostatic disorders, hemorrhages and acute renal failure [2,5,6,7,8]. However, the local and systemic pathophysiology caused by snake venom is not restricted to a condition mediated by the direct action of toxins but also by the body’s reaction to them. 

Based on the converging biochemical and physiological information, it was possible to determine which endogenous processes allow venoms to use the physiological machinery of the victim to increase toxicity [9]. The most important effects of the venom are based on endogenous processes in molecular cascades that suffer a disproportional activation causing positive feedback of coagulation, inflammation, and tissue degradation/remodeling, among other processes [10,11]. In this context, the term “thromboinflammation” has been used to describe the activation of the hemostatic system in cascade as well as the activation of blood cells that can contribute to increasing the toxicity of venoms [11,12].

Therefore, the aim of the present study is to investigate quantitative alterations in the plasma proteome of mice envenomated by *B*. *atrox*. This study was performed aiming to explore biological pathways that are simultaneously affected in murine organisms after the envenomation by *B*. *atrox*. Our studies depended on an exploration of the quantitative behavior of the murine plasma proteome. From the integration of the quantitative values of its components, it was possible to reveal the modification of biological pathways through proteins belonging to a category that underwent changes in their abundance. This allowed us to identify the signaling systems related to inflammation, hemostasis, multicellular blood system, and other disorders simultaneously, suggesting the establishment of thromboinflammation after *B*. *atrox* snakebite envenoming.

## 2. Results and Discussion

**Figure 1:**
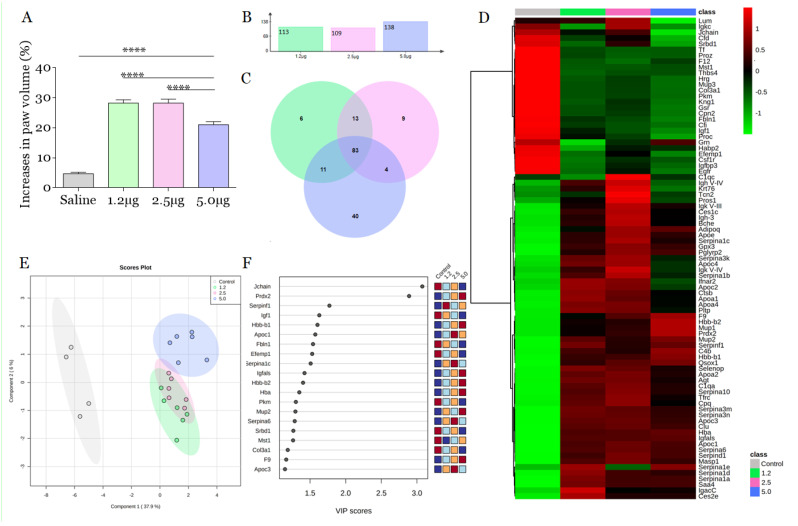
*B. atrox* venom induces edema and changes in the blood plasma proteome of mice. (**A**) Edematogenic profile of *B. atrox* venom by dose-response. *B. atrox* venom was injected i.pl. in the right hind paw of male swiss mice (*n* = 6). Paw edema 30 min after injection with venom with the aid of a digital caliper and expressed in % increase in relation to the initial thickness. The columns represent the mean ± SD (*n* = 6/group). **** *p* < 0.0005 for the comparisons indicated (one-way ANOVA). (**B**) Number of proteins with differential abundance (DAPs) (log-FC ≥ 1) identified in each group. (**C**) Venn diagram describing the distribution of DAPs among groups. (**D**) Heatmap shows the turnover of common plasma proteins (CPP) obtained from mice injected with 1.2, 2.5, and 5.0 μg of *B. atrox* venom. High protein abundance is shown in shades of red and low abundance is shown in shades of green. The abundance of each protein was normalized by the internal standard to generate a data matrix consisting of signal intensity values followed by scaling data centered on the mean and generalized logarithmic transformation. Values are presented as the mean of each experimental group. (**E**) Partial Least Squares Discriminant Analysis (PLS-DA) imp. features, *p* < 0.05 of the proteome of blood plasma mice (*n* = 6: three biological pools/replicas × two technical replicas). (**F**) The variable importance projection (VIP) plot displays the top 20 most important protein resources identified by the PLS-DA. The colored boxes on the right indicate the relative abundance of the corresponding protein for plasma samples from mice injected with venom. VIP is a weighted sum of squares of the PLS-DA loads taking into account the amount of Y variable explained in each dimension.

Edema is the most common inflammatory sign in *B. atrox* envenomation, and it can progress to a compartment syndrome resulting in tissue loss and amputations [13,14,15]. To investigate the early changes caused in the paw of animals induced by the venom of *B. atrox*, we carried out a study focused on the effects of the venom 30 min after injection. During this period, we focused on the direct action of the venom toxins, avoiding the interference of secondary effects of endogenous components released by the local reaction [12]. We verified that 1.2 μg of *B. atrox* venom resulted in an increase in paw volume of 28.2% ± 0.48 (*p* < 0.0001). Furthermore, 2.5 μg induced a response similar to 1.25 μg (28.20% ± 0.58, *p* < 0.0001), characterizing a constant response between these doses. On the other hand, 5.0 μg of *B. atrox* venom induced 21% ± 0.44 (*p* < 0.0001) of edema, lower when compared to previous doses (Figure 1A).

Here, we elucidate an important step for understanding the mechanisms involved in the systemic response against the severity of edema caused by *B. atrox* venom. For this purpose, plasma samples were inspected 30 min after the injection of three increasing doses of venom to assess the first events involved in the snake envenomation [16]. We found that *B. atrox* venom induced edema at all doses tested following the time of 30 min, according to studies that show the maximum development of edema in this period, followed by progressive decreases [17]. During this period, we focused on understanding the action of the snake venom toxins, avoiding the interference of the secondary effects of the endogenous components released by the local reaction or tissue repair system.

The proteome was based on protein intensity by the extracted ion chromatograms (XIC) (Supplementary Material Table S1), the number of unique peptides (≥1), and log-fold change (Supplementary Material Table S2), which generated a list with 113, 109 and 138 proteins of differentially abundance proteins (DAPs) in the plasma from mice after injection with 1.2, 2.5, and 5.0 μg of *B. atrox* venom, respectively (Figure 1B). In general, 83 common plasma proteins were changed in all groups regardless of the dose of venom used (Figure 1C,D). Further comparisons among experimental groups were detailed on a heat map. Compared to the control group, 27 proteins showed a reduction in their abundance after injection of *B. atrox* venom, while another 56 increased (Figure 1D). Various DAPs were identified in the plasma of mice injected with *B. atrox* venom, as well in another study using *B. leucurus* venom [12]. However, we found a difference in the number of DAPs for *B. atrox* envenomation when compared with the amount identified in the plasma of mice envenomed with the *B. leucurus* venom reported by Cavalcante and collaborators [12]. This difference may be related to the variability for edema caused by the same quantity of venom using these two venoms.

PLS-DA analysis has shown that the plasma proteome of the three experimental groups injected with different doses of venom differed from the control group, as well as in the edema quantification assay. The plasma proteome content of mice injected with 1.2 μg and 2.5 μg of *B. atrox* venom have shown an overlap, indicating a high degree of similarity regarding changes in protein abundance. Furthermore, the protein profile from the plasma of mice injected with 5.0 μg of *B. atrox* venom (Figure 1E) has shown a great dissimilarity when compared to the groups injected with 1.2 and 2.5 μg of venom. The classification of the patient’s clinical condition is based on signs and symptoms. Thus, the identification of proteins that discriminate between different classifications of the severity of envenomation can help in the management and therapeutic conduct.

**Figure 2:**
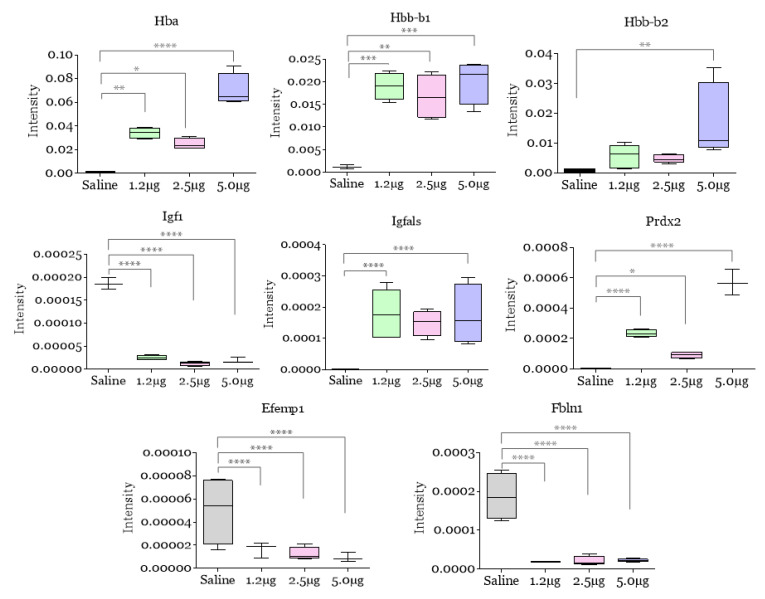
Comparative analysis of hemoglobin and subunits (Hba, Hbb-b1/b2), peroxiredoxin 2 (Prdx2), factor IX (F9), insulin-like growth factor 1 (Igf1), epidermal growth factor containing fibulin-like extracellular matrix protein 1 (Efemp1) and fibulin (Fbln1) level alterations in mice 30 min after intraplantar injection with *B. atrox* venom. Trends of a few selected differentially abundant proteins in blood plasma proteome alterations identified by mass spectrometry. Data are represented by the normalized intensity of proteins captured by an extracted ion chromatogram (XIC). The Y-axis of the box plots were normalized (log-fold) intensity. The columns represent the mean ± SD (*n* = 6/group). * *p* < 0.05, ** *p* < 0.01, *** *p* < 0.005, and **** *p* < 0.0005 for the comparisons indicated (one-way ANOVA).

We attempt to find the particularities of each group, generating a list of significant characteristics validated by VIP scores among experimental and control groups (Figure 1F). VIP scores have evidenced a dose-response among experimental groups in relation to the control: hemoglobin and subunits (Hba, Hbb-b1/b2), peroxiredoxin 2 (Prdx2), factor IX (F9), insulin-like growth factor 1 (Igf1), epidermal growth factor containing fibulin-like extracellular matrix protein 1 (Efemp1) and fibulin (Fbln1) can act as possible biomarkers of the lesion in *B. atrox* envenomation (Figure 2).

After the mice were envenomated by *B. atrox* venom, we found an increase in the presence of extracellular Hb, suggesting erythrocyte rupture, and this can be related to thrombotic microangiopathy, which is reported experimentally and clinically [18,19,20,21]. In addition, ecchymoses, as well as their process, also configure a mechanism for the release of extracellular hemoglobin (Hb). Hb induces programmed necrosis in macrophages [22] and maintenance of M1 macrophages due to the formation of intracellular iron storage from erythrocytes [23]. This scenario suggests that in an envenomation, this molecule may be a signal of positive feedback in tissue degradation caused by *B. atrox* venom, in addition to a likely role as a monitoring biomarker for tissue damage. However, alterations in Hb amount on blood counts were not found (Supplementary Material Figure S1). This discrepancy between detecting changes using these different methods is likely due to the fact that spectrometry detects and infers proteins in a sample more sensitively.

Moreover, Prdx2 is a negative regulator of hypoxia-inducible factors (HIF) and inhibits the transcriptional activity of STAT3 through redox effects [24,25]. Thus, the presence of increased levels of Prdx2 according to the quantity of venom in our study may be associated with edema-induced hypoxia. However, the origin of Prdx2 must be clarified, as thrombin can increase the levels of this protein since this molecule is the primary antioxidant in erythrocytes [26]. Interestingly, Prdx2 showed an increase in the proteome of mice injected with 5.0 µg of *B. atrox* venom. This protein can attenuate the activation of matrix metalloproteinases (MMPs) and reduce oxidative stress [27,28,29].

On the other hand, factor IX is a vitamin K-dependent zymogen activated by factor XIa/Ca++ (intrinsic pathway) or by factor VIIa/tissue factor/Ca++ (extrinsic pathway) associated with bleeding disorders. Our data showed an increase in this protein after injection with *B. atrox* venom. It is known that this experimental envenomation is classically marked by bleeding disorders, and in cases of systemic bleeding, there is the consumption of coagulation factors and fibrinolytic components, in addition to the participation of tissue factors [6]. However, Factor IX does not seem to be related to such phenomena and, probably, it may be associated with the inflammation caused by the *B. atrox* venom or even play a role up to now unknown in the pathophysiology of the envenomation. It is important to emphasize that these proteins can be indicators of the envenomation’s acute phase. Thus, it remains to be seen whether these preclinical markers identified in plasma for edema severity and thromboinflammation in our study are validated in larger preclinical cohorts as well as in clinical cohorts. The importance of this study is the possibility of greater variability among mice since pools were used for MS analysis.

The analysis of regulatory trends of the main altered biological processes in which common plasma proteins are involved was explored. Thus, there were 40 main altered biological processes classified into four main clusters: (i) processes related to the lipid system, (ii) immune systems and coagulation, (iii) multicellular blood system, and (iv) regulation of gene expression and cell cycle. 

**Figure 3:**
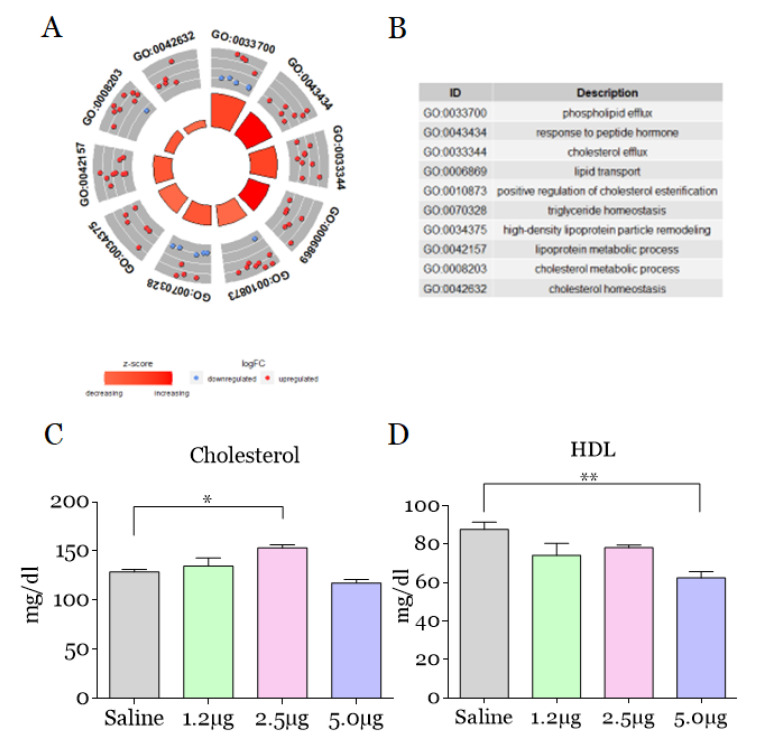
Principal biological processes related to the lipid system that enriched blood plasma of mice 30 min after intraplantar injection with *B. atrox* venom. (**A**) Identification of the main differentially regulated ontological (GO) gene terms, graphically displayed according to significance, with a measure of overall up- or down-regulation for category after experimental envenomation using *B. atrox* venom. The outer circle shows the number of proteins whose color of each protein corresponds to the logarithmic change, with red representing the increase in abundance and blue representing the decrease. The inner rectangles represent the *p*-value of the GO term and are colored according to the z-score to represent the general direction of change for each individual term. (**B**) The table lists the GO terms. (**C**–**D**) Cholesterol and HDL values in the serum of mice after injection with 1.2, 2.5, and 5.0 µg of *B. atrox*. The columns represent the mean ± SD (*n* = 6/group). * *p* < 0.05 and ** *p* < 0.01 for the comparisons indicated (one-way ANOVA).

The processes related to the lipid system were up-regulated, namely: efflux, response to peptide hormone, cholesterol efflux, and lipid transport. Furthermore, alterations related to positive regulation of cholesterol esterification, triglyceride homeostasis, high-density lipoprotein particle remodeling, lipoprotein metabolic process, cholesterol metabolic process, and cholesterol homeostasis were also evidenced (Figure 3A,B). In this study, we verified changes in lipid homeostasis after experimental envenomation. It is known that phospholipase A_2_ (PLA_2_) can induce the formation of lipid droplets in macrophages [30]. *B. moojeni* venom induces the release of prostaglandin E2 in pre-adipocytes [11], while the *B. atrox* venom induces local inflammation mediated by the synthesis and substantial release of several inflammatory mediators [31], which supports the enrichment related to the efflux of lipids pointed here.

Our results suggest that *B. atrox* venom induces overregulation of lipid processes that would be related to an increase in lipid levels in plasma after injection of venom. Then, we investigated possible changes in cholesterol, triglycerides, HDL, LDL, and VLDL levels in mice plasma. Our findings have ratified the changes observed in cholesterol and HDL levels in mice injected with 2.5 and 5.0 µg of *B. atrox* venom, respectively (Figure 3C,D). However, changes in LDL and VLDL triglyceride levels were not identified (Supplementary Material Figure S2). Once again, this divergence can be due to the sensitivity differences between methods used. Spectrometry detects and infers proteins in a sample more sensitively, while GO analysis classifies and frames them in the ways in which these proteins are functional and biochemical assays analyze the concentrations of lipid markers. However, it is clear that lipid mediators are potent immune response stimulators [32,33], and thus, cholesterol and HDL may be related to the severity of the envenomation.

**Figure 4:**
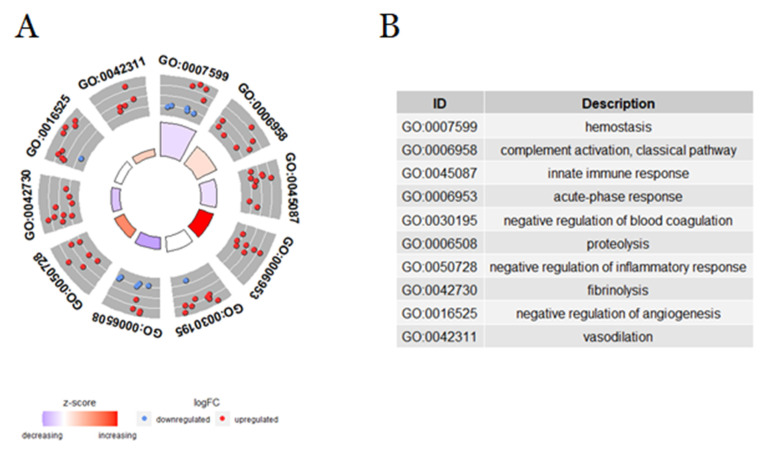
Principal biological processes related to immune systems and coagulation that were enriched in the blood plasma of mice 30 min after intraplantar injection with *B. atrox* venom. (**A**) Identification of the main differentially regulated ontological (GO) gene terms, graphically displayed according to significance, with an overall up- or down-regulation measure for the category after injected with *B. atrox* venom. The outer circle shows the number of proteins whose color of each protein corresponds to the logarithmic change, with red representing the increase in abundance and blue representing the decrease. The inner rectangles represent the *p*-value of the GO term and are colored according to the z-score to represent the general direction of change for each individual term. (**B**) The table lists the GO terms.

The cluster of biological processes related to the immune and coagulation systems showed particular regulatory tendencies. Hemostasis, innate immune response, proteolysis, and fibrinolysis were down-regulated in mouse plasma after *B. atrox* envenomation in our study. On the other hand, the activation of the classical pathway from the complement system, acute-phase response, negative regulation of inflammatory response, and vasodilation have presented an up-regulation. Furthermore, although DAPs were found to be related to the terms GO-negative regulation of blood coagulation and negative regulation of angiogenesis, these processes remained neutral when analyzed (Figure 4). It is important to know that such alterations in hemostatic and immunological processes could cause significant systemic disturbances.

**Figure 5:**
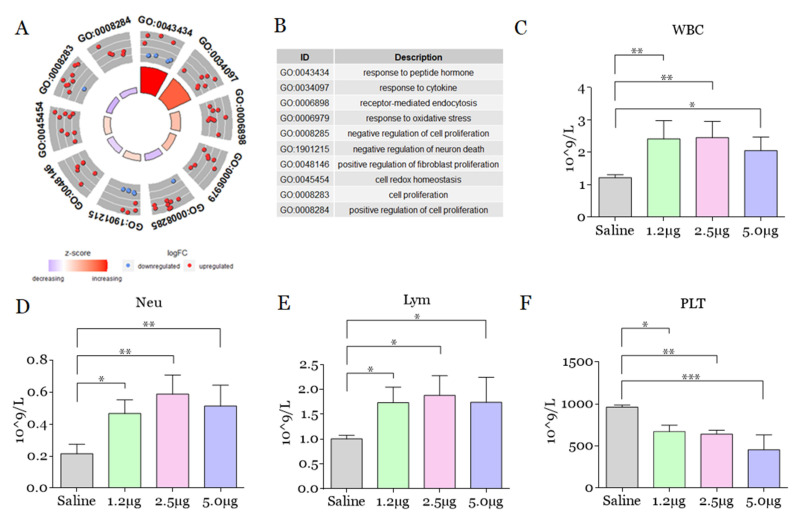
Principal biological processes related to blood cells and in mouse leukocyte and platelet counts 30 min after intraplantar injection with *B. atrox* venom. (**A**) Identification of the main differentially regulated ontological gene (GO) terms, graphically displayed according to significance, with an overall measure of up- or down-regulation for the category after injection with *B. atrox* venom. The outer circle shows the number of proteins whose color of each protein corresponds to the logarithmic change, with red representing the increase in abundance and blue representing the decrease. The inner rectangles represent the *p*-value of the GO term and are colored according to the z-score to represent the general direction of change for each individual term. (**B**) The table lists the GO terms. (**C**–**F**) Count of circulating total leukocytes (WBC), neutrophils (Neu), lymphocytes (Lym), and platelets (PLT) in the blood of mice 30 min after injection with 1.2, 2.5, and 5.0 µg of *B. atrox*. The columns represent the mean ± SD (*n* = 6/group). * *p* < 0.05, ** *p* < 0.01 and *** *p* < 0.005 for the comparisons indicated (one-way ANOVA).

Regarding the categories involved with the multicellular blood system, response to peptide hormone and response to cytokine were the processes with the greatest change (up-regulation), although receptor-mediated endocytosis, response to oxidative stress, negative regulation of neuron death, and cell redox homeostasis were also up-regulated. In contrast, *B. atrox* venom induces attenuation in the ratio of negative and positive regulation of cell proliferation and positive regulation of fibroblast proliferation (Figure 5). As our findings showed that *B. atrox* venom produces alterations in biological processes in the plasma of mice related to homeostasis/cellular response, we proceeded with blood cell counts in these mice to confirm the alterations in the multicellular blood system. We identified leukocytosis among all groups injected with *B. atrox* venom compared to the control group (Figure 5C). In addition, neutrophilia (Figure 5D) and lymphocytosis (Figure 5E) were found in mice injected with venom. In addition, all mice had thrombocytopenia (Figure 5F), but changes in the red blood series were not observed (Supplementary Material Figure S2).

Thus, platelet alterations were confirmed through the circulating platelet count after envenomation. In all cases, thrombocytopenia was present, supporting the previous findings regarding the alteration in the abundance of proteins related to platelet processes. Thrombocytopenia is a phenomenon already reported in patients after snakebite envenoming by *B. atrox* [6]; however, the role of platelets and if there is sequestration, apoptosis, or other phenomena that justifies the reduction in platelet count in the snakebite envenomation by this species is still unknown [2,34,35].

Various toxins, especially PLA_2_, isolated from *Bothrops* venoms have already been cataloged with the potential to inhibit or induce platelet aggregation. In the case of B. atrox, Batroxhragin [36], Atroxlysin I and III [37,38], thrombocytin [39], and BatroxLAAO [40] are the known toxins that act on platelets, which could justify the degranulation of platelets, an event found in this study. However, the presence of signaling proteins for platelet degranulation suggests that thrombocytopenia may be an effect induced by secondary mechanisms during envenomation, not by the direct action of toxins.

Our data set evidenced a heightened neutrophil degranulation, suggesting its early activation during the envenomation. Neutrophils are present in myonecrotic and hemorrhagic areas and in inflammatory infiltrate [41], and once activated, they produce proinflammatory cytokines, phagocytize and release neutrophil extracellular traps (NETs), acting in tissue repair [42,43,44,45,46,47,48]. Here, we identified that mice showed neutrophilia after experimental envenomation, as also described in clinical findings, wherein neutrophilia of variable intensity has been reported in *Bothrops* envenomation, especially with greater intensity in cases with tissue loss and/or limb amputations [13,14,15]

**Figure 6:**
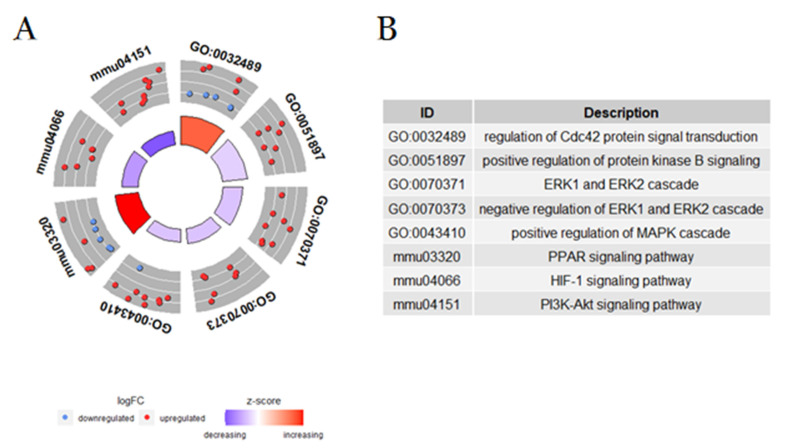
Principal signaling pathways related to regulation of gene expression and enriched cell cycle in blood plasma of mice 30 min after intraplantar injection with *B. atrox* venom. (**A**) Identification of the main differentially regulated ontological gene terms (GO), graphically displayed according to significance, with an overall up- or down-regulation measure for the category after injection with the *B. atrox* venom. The outer circle shows the number of proteins whose color of each protein corresponds to the logarithmic change, with red representing the increase in abundance and blue representing the decrease. The inner rectangles represent the *p*-value of the GO term and are colored according to the z-score to represent the general direction of change for each individual term. (**B**) The table lists the GO terms.

We also found up-regulation of Cdc42, which suggests its role in the immune response induced by *B. atrox* venom (Figure 6). It is already known that activation of Cdc42 is important for motility and directionality of neutrophil migration [49,50], regulating ROS formation, degranulation, and neutrophil activation in a stimulus-dependent manner [51]. T-cell homeostasis (limitation and proliferation) restricts Th1 and Th17 cell differentiation via glycolysis repression while inducing Th2 and iTreg differentiation and nTreg cell homeostasis and stability by cell stability iTreg and nTreg through induction of glycolysis [52].

On the other hand, we verified the attenuation of the down-regulation via ERK1/2 and of the up-regulation of MAPK. ERK1/2 is a member of the “generic” mitogen-activated protein kinase (MAPK) signaling pathway [53]. The ERK1/2 signaling pathway plays a crucial role in cell proliferation and gene expression, impacting the cell cycle, cell migration, cell invasion [54], apoptosis [55], autophagy [56,57], cell metabolism [58], and inflammation [59]. However, MAPK signaling regulates many cellular events, such as cell proliferation, differentiation, cell migration, controlled cell death (apoptosis), and senescence, as well as certain aspects of cell cycle progression, cell survival, metabolism, and transcription [60]. The suppression of down-regulation of the ERK1/2 pathway may suggest that cellular pathways related to the cell cycle and cell death are overexpressed. Furthermore, the attenuation of MAPK up-regulation suggests an attempt to control cell death, probably those cells injured at the site of injection of *B. atrox* venom.

Although a serine proteinase from *B. atrox* venom induces angiogenesis through the PI3K/Akt signaling axis [61], we found an attenuation in the PI3K/Akt and HIF-1 pathways after *B. atrox* envenomation. Since the discovery of the HIF-1 pathway, several studies have been carried out to understand the role of this pathway in various diseases [62]. However, it is the first time that a possible role of this pathway has been demonstrated in snakebite envenoming, although it is known that HIF-1 is responsible for signaling adaptive responses in the absence of oxygen that provide ischemic tolerance, causing up-regulation of transcriptional cascades for tissue protection and adaptation [62,63] and thus, this pathway may be related to tissue plasticity against ischemia caused by the *B. atrox* venom.

**Figure 7:**
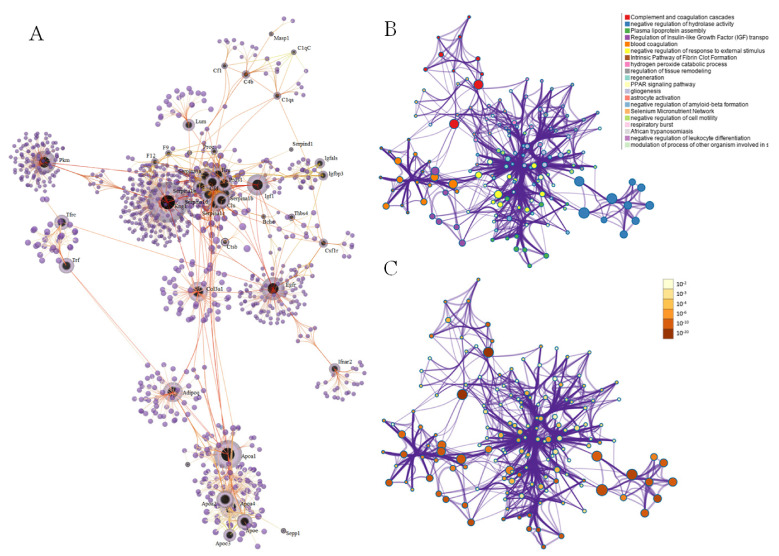
Network of protein-protein interactions and major cluster of altered processes blood plasma of mice 30 min after intraplantar injection with *B. atrox* venom. (**A**) Main proteins of the iteration network. (**B**) Network of enriched terms colored by cluster ID, where nodes that share the same cluster ID are typically close to each other. (**C**) Network of enriched terms colored by *p*-value, where terms containing more genes tend to have a more significant *p*-value.

After injection of *B. atrox* venom, processes related to coagulation, complement, and tissue spatial arrangement were enriched (Figure 7A). Enrichment analysis identified clusters in which DAPs were associated with complement and coagulation cascades, blood coagulation, and intrinsic pathway of fibrin clot formation (Figure 7B,C). Based on the association between envenoming and the set of immune-related DAPs, we further explored the immune story. Several DAPs have been linked to immune system processes: regulation of tissue remodeling, regeneration and hydrogen peroxide catabolic process (Supplementary Material Table S3).

We found evidence of down-regulation in the abundance of epidermal growth factor receptor (Egfr), a maintenance regulator of oxidative stress in macrophages, macrophage infiltration and induction of proinflammatory cytokines, downstream activation of transcription factors, as the nuclear factor-κB (NF-κB) [61,62,63,64,65,66,67,68]. These findings likely reflect a compensatory response to counteract the rise in reactive oxygen species during *B. atrox* envenomation.

## 3. Conclusions

Our results have revealed that plasma proteome is modulated according to the amount of edema induced by the *B. atrox* venom. Differential abundance of some proteins (Hba, Hbb-b1/b2, Prdx2, F9, Igf1, Efemp1, and fibulin) have suggested that these molecules can be potential indicators of the severity of edema/inflammation in mice caused by *B. atrox* venom to be tested in futures studies. However, monitoring the quantitative tendencies of these proteins based on the course-time of envenomation was beyond the scope of this study. This leaves a lack of information about certain key envenomation responses during the regulation of murine proteins in the *B. atrox* venom response. These could be an effective future continuation of the present investigation, especially as the results continue to advance for translational research. Collectively, our findings have provided some new mechanistic insights into the evolution of *B. atrox* envenomation, and we anticipate that this will accelerate opportunities for the development of clinical trials based on the envenomed organism’s proteins to monitor the severity and complexity of envenomation, not only by *B. atrox* but also by other species of snakes and other venomous animals.

We also underscore the view that the venom-induced pathophysiology of *B. atrox* results from a combination of the direct action of venom toxins and indirect mechanisms derived from the tissue inflammatory response to envenomation. Differences in tissue inflammatory responses of this venom may contribute to variations in the pathophysiological scenario, including thromboinflammatory alteration in lipid metabolism and disturbances in the cell state characterized by oxidative stress, as well as effects on pathways that regulate the survival and cell cycle and gene expression.

## 4. Materials and Methods

### 4.1. Snake Venom Bothrops atrox

*B. atrox* venom pool was obtained from 29 specimens donated to the Center for the Study of Venoms and Venomous Animals (CEVAP) at Sao Paulo State University (UNESP) under authorization from the Brazilian Institute for the Environment and Renewable Natural Resources (IBAMA 1/35/92/0044-1, Proc. No. 02001.005670/90-77). This research project is registered at the National System of Genetic Heritage and Associated Traditional Knowledge (SISGEN) under nº A54E8E8. The snakes were anesthetized with the aid of carbon dioxide (CO_2_), and the venom extracted, diluted in 0.9% (*m/v*) saline, filtered with 0.45 µm pore support, and centrifuged at 8000× *g* for 15 min. The supernatant was collected, lyophilized, and stored at −20 °C in a pool until the moment of use [69].

### 4.2. Animals and Experimental Envenomation

Male Swiss mice (*n* = 6/group) were injected with either 1.2 µg, 2.5 µg, or 5.0 µg of *B. atrox* venom and 0.9% (*m/v*) sterile saline via intraplantar. The individual thickness of the right hind paw was measured before the venom injection (baseline) and 30 min after the edema induction using a digital caliper (Digimess, São Paulo, SP, Brazil). The edema was expressed as the percentage difference between the paw thickness after and before the venom injection calculated with the following formula: (Te−T0) / T0 × 100. The statistical analysis was performed using the *t*-test, one-way ANOVA, followed by the Tukey test of multiple comparisons (*p* ≤ 0.05) and expressed as mean and standard deviation.

### 4.3. Hematologic and Lipidic Analysis

For hematological analysis, new groups of mice were injected with *B. atrox* venom as described above, and blood was obtained by cardiac puncture. Blood samples containing EDTA were analyzed in an automated hematology counter BC-5000 (Mindray^®^). Measured parameters included red blood cell count (RBC), hemoglobin (HGB), hematocrit (HCT), mean corpuscular volume (MCV), mean corpuscular hemoglobin (HCM), mean corpuscular hemoglobin concentration (MCHC), white blood cell count (WBC), neutrophils (Neu), lymphocytes (Lym) and platelets (PLT). In addition, serum samples were used to measure cholesterol, triglycerides, high-density lipoprotein cholesterol (HDL), low-density lipoprotein cholesterol (LDL), and very low-density lipoprotein cholesterol (VDL). The statistical analysis was performed using the *t*-test, one-way ANOVA, followed by the Tukey test of multiple comparisons (*p* ≤ 0.05) and expressed as mean and standard deviation.

### 4.4. Collection, Selection, and Grouping of Plasma Samples

After general anesthesia with ketamine (80 mg/kg, ip) and xylazine (8 mg/kg, ip), the mice were bled via cardiac puncture, and the blood was stored in EDTA tubes and centrifuged at 5000 rpm for 10 min at 4 °C. In case of hemolysis or contamination by residues from other organs, the samples were discarded. Pools were formed with 10 µL of plasma from two animals, which resulted in three pools per group.

### 4.5. LC-MS/MS Analyses and Data Processing

For the LC-MS/MS analysis, depletion strategies were not used due to the possibility that this strategy could remove important proteins together with the major ones. Plasma proteins (30 µg) were reduced, alkylated, and digested in order to obtain peptides, according to Cavalcante and collaborators (2022) [12]. Peptides were separated with a nano-ultra-performance by an Ultimate 3000 LC (Dionex, Germering, Germany). The mobile phases were 0.1% FA in water (A) and 0.1% FA in 100% acetonitrile (B). We used an isocratic gradient of 2% B 40 35% B in 120 min (300 nL/min) and eluted by the Reprosil-Pur C18-AQ analytical column, 3 µm, 120 Å, 105 mm (PICOCHIP, New Objective), followed by 55% to 90% B for 1 min, maintained to 90% B for 10 min and rebalanced to 2% B for 10 min. Nanospray ESI-MS was performed on a Thermo Q Exactive high-resolution mass spectrometer (Thermo Scientific, Waltham, MA, USA) according to Cavalcante and collaborators (2022) [12]. Raw MS files were obtained by Thermo Xcalibur software (version 4.0.27.19, ThermoFisher Scientific Inc.) and submitted to PatternLab for Proteomics 4 for protein identification and quantification [70]. For this purpose, a FASTA file with proteins corresponding to the reviewed SwissProt entries for the *Mus musculus* proteome from Uniprot was used (UP000000589). Protein-level and peptide-level FDR were ≤1. The files containing protein intensity, obtained by the extracted ion chromatograms (XIC) of peptides, were normalized and centered by average, and the fold change values (Log-Fc) and *p*-values were calculated using the *t*-test of Student using MetaboAnalyst. Then, the individual lists by a group of validated proteins were analyzed by Jveen to obtain the distribution of proteins between groups. Proteins that were common to all groups were analyzed by PLS-DA and Vip Score using MetaboAnalyst to identify the proteins responsible for the difference between those groups. The reported data were FDR-adjusted *p*-values. All proteins were analyzed according to ontological gene annotations using DAVID, and the results were plotted using GoPlot. Finally, proteins were also analyzed for their interactions using NetworkAnalyst to elucidate key proteins among the network of interactions and by Metascape to analyze enriched clusters and physical interactions between proteins.

## Data Availability

The mass spectrometry data in this manuscript has been uploaded to the MassIVE Repository from the Computer Science and Engineering University of California, San Diego (ftp://massive.ucsd.edu/MSV000089455/, accessed on 10 May 2021) with the data set identifier MSV000089455.

## Supplementary Materials

The following supporting information can be downloaded at: https://www.mdpi.com/article/10.3390/toxins14090613/s1, **Figure S1:** Hematological analysis analyzed in an automated hematology counter BC-5000 (Mindray^®^). Measured parameters in the mice serum plasma included red blood cell count (RBC), hemoglobin (HGB), hematocrit (HCT), mean corpuscular volume (MCV), mean corpuscular hemoglobin (MCH), mean corpuscular hemoglobin concentration (MCHC), red cell distribution coefficient of variation (RDW-CV), and red cell distribution standard deviation (RDW-SD). The columns represent the mean ± SD (*n* = 6/group). ** *p* < 0.01 and **** *p* < 0.0005 for the comparisons indicated (one-way ANOVA). **Figure S2:** Triglycerides, low-density lipoprotein cholesterol (LDL), and very low-density lipoprotein cholesterol (VLDL) levels were measured in the mice serum plasma. The columns represent the mean ± SD (*n* = 6/group). **Table S1:** Protein quantification based on protein intensity by the extracted ion chromatograms (XIC). **Table S2:** Proteins with differentially significant abundance by log-fold change. **Table S3:** Proteins interaction by enrichment analysis.
