# Supplementary material for: Experimental Bothrops atrox Envenomation: Blood Plasma Proteome Effects after Local Tissue Damage and Perspectives on Thromboinflammation
Source: Toxins (Basel). 2022 Sep 1;14(9):613. doi: 10.3390/toxins14090613 (PMC9503785; doi:10.3390/toxins14090613)
Supplement: Supplementary file 1 [file toxins-14-00613-s001.zip › Supplementary Material Figure S1.pdf]

toxins

MDPI

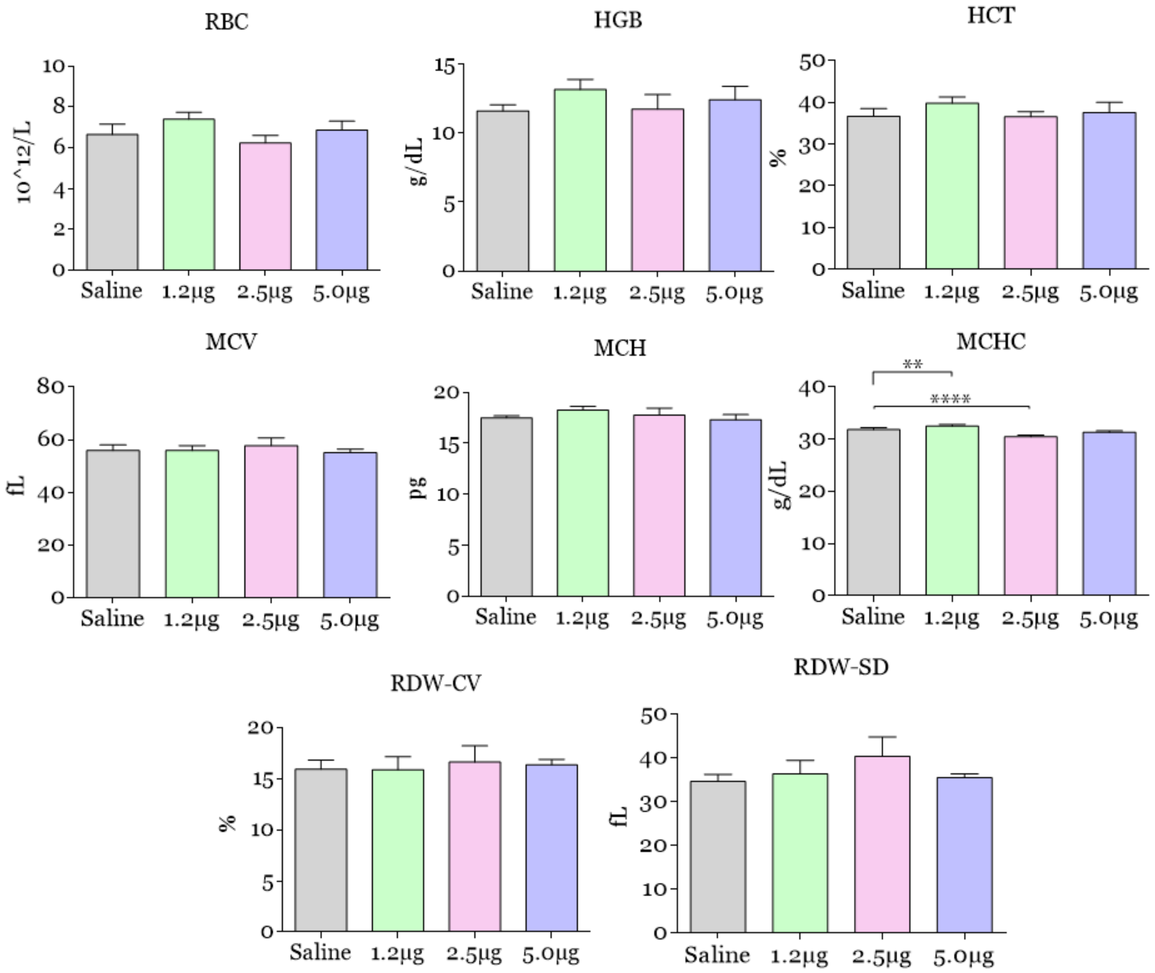

Figure S1: Hematological analysis analyzed in an automated hematology counter BC-5000 (Mindray®). Measured parameters in the mice serum plasma included red blood cell count (RBC), hemoglobin (HGB), hematocrit (HCT), mean corpuscular volume (MCV), mean corpuscular hemoglobin (MCH), mean corpuscular hemoglobin concentration (MCHC), Red Cell Distribution-Coefficient of Variation (RDW-CV) and Red Cell Distribution-Standard Deviation (RDW-SD). The columns represent the mean ± SD (n = 6/group). **p < 0.01 and ****p < 0.0005 for the comparisons indicated (one-way ANOVA).
