# Supplementary material for: Experimental Bothrops atrox Envenomation: Blood Plasma Proteome Effects after Local Tissue Damage and Perspectives on Thromboinflammation
Source: Toxins (Basel). 2022 Sep 1;14(9):613. doi: 10.3390/toxins14090613 (PMC9503785; doi:10.3390/toxins14090613)
Supplement: Supplementary file 1 [file toxins-14-00613-s001.zip › Supplementary Material Figure S2.pdf]

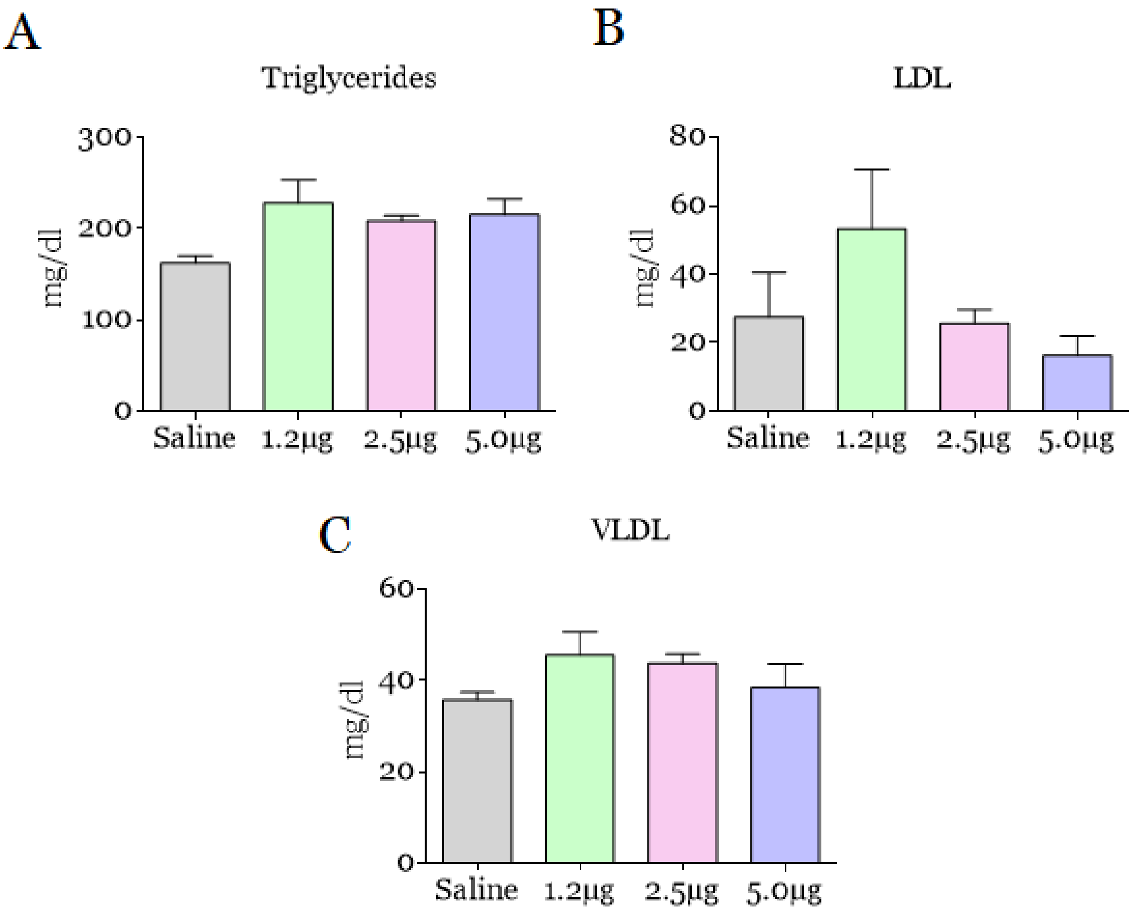

Figure S2: Triglycerides, low density lipoprotein cholesterol (LDL) and very low-density lipoprotein cholesterol (VLDL) levels were mensuared in the mice serum plasma. The columns represent the mean ± SD (n = 6/group).
